# Caring for Patients with Gestational Hypertensive Disorders: Essential Takeaways

**DOI:** 10.14797/mdcvj.1311

**Published:** 2024-03-14

**Authors:** Paola Gomez-Aviles, Alfredo F. Gei, Pavel Martinez-Dominguez

**Affiliations:** 1 National Institute of Medical Sciences and Nutrition Salvador Zubiran, Mexico City, Mexico; 2 Houston Methodist Hospital, Houston, Texas; 3 Houston Center for Maternal Fetal Medicine, Houston, Texas, US; 4 National Institute of Cardiology Ignacio Chavez, Mexico City, Mexico

**Keywords:** hypertensive disorders in pregnancy, gestational hypertension, preeclampsia, HELLP syndrome

## Abstract

Hypertensive disorders in pregnancy (HDP) are a group of conditions—including chronic hypertension, gestational hypertension, preeclampsia with and without end-organ damage, and acute complications, which include HELLP (hemolysis, elevated liver enzymes, and low platelets) syndrome and eclampsia—that could lead to severely adverse outcomes for both mother and fetus. The incidence of HDP has increased, affecting one out of seven delivery hospitalizations. Physicians should be aware of HDP for early identification and proper treatment to improve patient outcomes.

## Introduction

Hypertensive disorders in pregnancy (HDP) are a group of conditions that can complicate the course of a pregnancy. Their incidence has increased as related risk factors such as obesity, gestational diabetes, and advanced maternal age have become more frequent.^1^ HDP affect one out of seven delivery hospitalizations and are currently the principal cause of pregnancy-related death in developed countries.^2^

According to the American College of Obstetricians and Gynecologists, HDP can be classified as:

- **Chronic hypertension:** Blood pressure (BP) ≥ 140/90 mm Hg before 20 weeks of gestation (WG) or hypertension persistent beyond 12 weeks after delivery.
- **Gestational hypertension:** BP ≥ 140/90 mm Hg after 20 WG in individuals who were previously normotensive.
- **Preeclampsia:** Systolic BP > 140 mm Hg and/or diastolic BP > 90 mm Hg after 20 WG in a woman who was normotensive at baseline and develops proteinuria or thrombocytopenia, increased transaminase levels, renal insufficiency, pulmonary edema, or new-onset headache (target organ involvement).
- **Chronic hypertension with superimposed preeclampsia:** History of chronic hypertension with a new onset of proteinuria, thrombocytopenia, impaired renal or liver function. Possible acute complications of HDP include eclampsia (new-onset seizures in a patient with preeclampsia) or HELLP syndrome (hemolysis, liver dysfunction, and low platelets).

**Figure:**
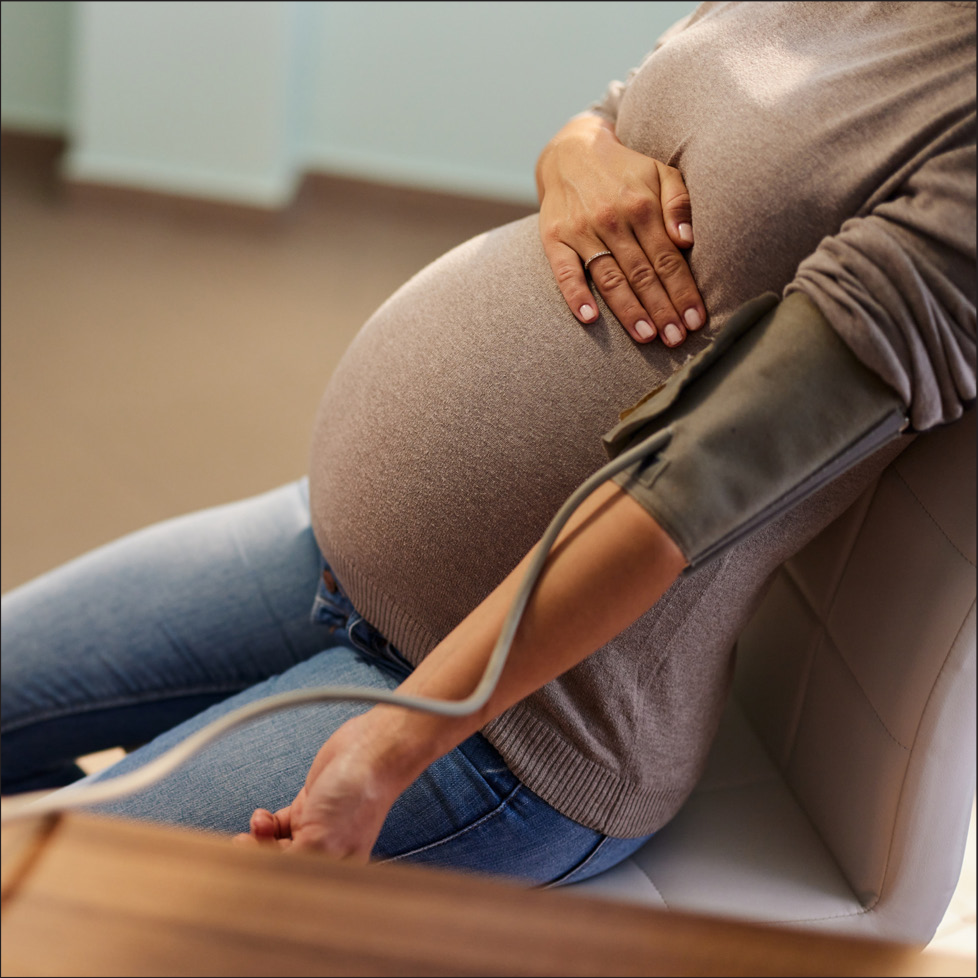


The following are points to remember in patients with HDP:

- **Preconception recommendations:** Identify modifiable risk factors that can be managed before pregnancy to reduce the risk of HDP. Women may benefit from weight counseling, glycemic control, and dietary and/or lifestyle modifications. If the patient has chronic hypertension, BP control should be optimized, and the physician should recommend appropriate lifestyle modifications such as avoiding excessive salt intake and caffeine.^3^ Also, during the preconception period, pregnancy-safe medication should be assessed and specific medications must be avoided, which include anticonvulsants (valproic acid, phenobarbital), antihypertensives (angiotensin-converting enzyme inhibitors, angiotensin receptor blockers), vitamin A and derivatives (isotretinoin), and lithium, among others.^4^
- **Accurate BP measurement:** Use a proper-sized cuff and place it directly over the patient’s arm, without clothing or rolling of the sleeves. The patient should have an empty bladder, be at rest for 5 minutes, and avoid caffeine or nicotine-containing products 30 minutes prior to the measurement. In addition, the patient should be sitting in a chair with a back and arm support, with her arm at the level of the heart. Feet should lay flat on the floor with legs uncrossed.^1^
- **Early diagnosis with baseline screening:** All pregnant women should be evaluated in the first trimester to provide an early diagnosis. According to the International Federation of Gynecology and Obstetrics, the baseline screening should evaluate mean arterial pressure and maternal risk factors. Other biomarkers useful for screening can be performed during the 11 to 13 weeks of gestation. These include uterine artery pulsatility index, serum pregnancy associated plasma protein-A, and serum placental growth factor. The risk factor calculator considers various parameters that include pregnancy type (singleton or twins), pregnancy dating, maternal characteristics such as racial origin, body mass index, smoking during pregnancy, medical and obstetric history, and biophysical measurements. The calculator is available at: https://fetalmedicine.org/research/assess/preeclampsia.^5^
- **Correct diagnosis according to HDP classification:** Diagnosis should be based on HDP classifications, which include chronic hypertension, gestational hypertension, preeclampsia, and chronic hypertension with superimposed preeclampsia.^1^
- **Recognize common red flags:** Identify acute, severe, and persistent BP elevations that are not justified by other causes. In women with chronic hypertension, it is important to identify transient BP increases from superimposed preeclampsia. It is recommended that physicians assess changes in symptoms and start evaluation with a complete blood count and creatinine and liver function tests to rule out complications including end-organ damage and/or HELLP syndrome.^3^
- **Use of low-dose aspirin:** Recommend a daily dose of 81 to 162 mg to women at high risk of preeclampsia, starting between 12 to 28 WG (ideally before 16 weeks). High-risk groups include patients with a history of preeclampsia, multifetal gestation, chronic hypertension, diabetes, renal disease, or autoimmune conditions.^6,7^
- **Initiation of treatment:** According to the American College of Obstetricians and Gynecologists (ACOG), tight control of BP has no benefits over the fetus, so antihypertensive therapy is recommended when BP is above 160/110 mm Hg. However, organizations have established different thresholds for initiating treatment; the International Society for the Study of Hypertension in Pregnancy recommends starting therapy at 140/90 mm Hg. As such, lower thresholds can be considered in patients with comorbidities or underlying impaired renal function.^1,5^ A recent practice-changing CHAP trial done in 2022 demonstrated that pregnant women treated with a BP target of < 140/90 mm Hg were associated with better pregnancy outcomes than individuals who did not receive antihypertensive therapy or were initiated at a higher threshold of > 160 mm Hg.^8^
- **Treatment plan:** β-blockers and calcium channel blockers, including labetalol and nifedipine, respectively, are the first-line treatment for HDP. Second-line treatment includes hydralazine and thiazide diuretics; however, administration of diuretics should be carefully monitored as they can impact intravascular volume and placental perfusion. In acutely severe hypertension, intravenous labetalol or hydralazine can be used.^5^ An intravenous magnesium sulfate infusion is used for seizure prophylaxis in severe cases. The use of angiotensin-converting enzyme inhibitors, angiotensin receptor blockers, and directrenin inhibitors are contraindicated during pregnancy due to significant fetal risks, such as teratogenicity and growth restriction. The definitive treatment in refractory hypertension is delivery of the fetus.^1^
- **Postpartum recommendations:** Within 2 weeks after delivery, patients can present with physiological high BP. Ambulatory BP surveillance may be prudent, mainly in patients who developed complications before or during pregnancy. Although rare, severe hypertension or preeclampsia may develop in the postpartum period, usually in women with antenatal hypertensive disorders, although it also can develop de novo.^3^ Postpartum preeclampsia should be considered with new-onset hypertension with the presence of severe features 48 hours to 6 weeks postpartum. Individuals with this clinical presentation require further evaluation—which should include medical history and a thorough physical exam—to address the underlying cause and complications. Laboratory evaluation should include electrolyte and renal function, platelet count, liver enzymes, and urine protein. Further imaging studies should be oriented towards the clinical presentation.

When the diagnosis is confirmed and other etiologies of hypertension are excluded, ACOG recommends starting management with the usual medications used during pregnancy, the first-line being IV labetalol or hydralazine and oral nifedipine. Unfortunately, there are no standardized management guidelines for specific medication titration in the postpartum period, therefore therapy should be individualized.^9^
